# Genotyping of Methicillin Resistant *Staphylococcus aureus* Strains Isolated from Hospitalized Children

**DOI:** 10.1155/2014/314316

**Published:** 2014-10-22

**Authors:** Mouna Ben Nejma, Abderrahmen Merghni, Maha Mastouri

**Affiliations:** ^1^Laboratoire des Maladies Transmissibles et Substances Biologiquement Actives “LR99ES27”, Faculté de Pharmacie de Monastir, Avenue Avicenne, 5000 Monastir, Tunisia; ^2^Laboratoire de Microbiologie, CHU Fattouma Bourguiba, 5000 Monastir, Tunisia

## Abstract

Community associated methicillin resistant *Staphylococcus aureus* (CA-MRSA) is an emerging pathogen increasingly reported to cause skin and soft tissue infections for children. The emergence of highly virulencet CA-MRSA strains in the immunodeficiency of young children seemed to be the basic explanation of the increased incidence of CA-MRSA infections among this population. The subjects of this study were 8 patients hospitalized in the Pediatric Department at the University Hospital of Monastir. The patients were young children (aged from 12 days to 18 months) who were suffering from MRSA skin infections; two of them had the infections within 72 h of their admission. The isolates were classified as community isolates as they all carried the staphylococcal cassette chromosome *mec* (SCC*mec*) IV and *pvl* genes. Epidemiological techniques, pulsed-field gel electrophoresis (PFGE) and multilocus sequence typing (MLST), were applied to investigate CA-MRSA strains. Analysis of molecular data revealed that MRSA strains were related according to PFGE patterns and they belonged to a single clone ST80. Antimicrobial susceptibility tests showed that all strains were resistant to kanamycin and 2 strains were resistant to erythromycin.

## 1. Introduction

Methicillin resistant* Staphylococcus aureus* (MRSA) was initially reported as a nosocomial pathogen responsible for adult infections [1]. However, MRSA strains have emerged in the community causing community-acquired infections. CA-MRSA has been recognized as a pathogen in adults and children without traditional risk factors for MRSA acquisition. Children colonized with MRSA are potential reservoirs for the spread of MRSA in the community [2, 3]. Furthermore, the infants and newborns with immunological immaturity, especially those born prematurely and those requiring specialized care, remained the major group susceptible to CA-MRSA infections.

Most CA-MRSA strains were associated with skin and soft tissue infections (SSTI) and necrotizing pneumonia [4]. The incidence of pediatric SSTI has increased rapidly in the previous decade [5–7].

Notably, community isolates differ significantly from nosocomial strains by the antimicrobial pattern and virulence profile. It is known that resistance to beta-lactams is mediated by the* mec*A gene carried by a mobile genetic element called staphylococcal cassette chromosome* mec* (SCC*mec*). CA-MRSA have been described as strains harboring the SCC*mec* type IV, type V, or type VII [8, 9] and remained susceptible to the majority of antimicrobial agents other than beta-lactams. Furthermore, these strains have been found to carry virulence genes encoding a leukocyte-killing toxin called the Panton-Valentine leukocidin (PVL) determinant [4].

In this report, we characterize clinical MRSA strains isolated from children hospitalized in the Pediatric Department at the University Hospital of Monastir, Tunisia. We are interested to investigate the phenotypic and genotypic markers of these isolates including antimicrobial resistance, SCC*mec* type,* pvl* genes, pulsed-field gel electrophoresis (PFGE) patterns, and multilocus sequence typing (MLST) of seven unlinked housekeeping genes (*arc*C,* aro*E,* glp*F,* gmk*,* pta*,* tpi*, and* yqil*).

## 2. Materials and Methods

### 2.1. Bacterial Strains

Eight MRSA strains were collected from clinical specimens of hospitalized children in the Pediatric Department at the University Hospital of Monastir, Tunisia, during a three-month period (from June to August 2013). The subjects were 6 boys and 2 girls, aged from 12 days to 18 months. The isolates were associated with skin infections: cutaneous abscesses (7 cases) and facial cellulites (1 case).

### 2.2. Identification

*S. aureus* were identified according to standard bacteriological procedures: Gram strain reactions, colony morphology, catalase, the ability to coagulate the rabbit plasma, and latex agglutination test (Bio-Rad).

### 2.3. Antimicrobial Susceptibility Tests

Susceptibility to the following antibiotics penicillin G, cefoxitin, moxalactam, kanamycin, amikacin, tobramycin, gentamicin, erythromycin, lincomycin, tetracycline, pristinamycin, furans, ofloxacin, trimethoprim-sulfamethoxazole, rifampicin, fusidic acid, fosfomycin, mupirocin, high mupirocin, vancomycin, teicoplanin, and linezolid was determined according to the recommendations of the Committee for Antimicrobial Testing of the French Society of Microbiology (CASFM) (http://www.sfm-microbiologie.org/) [10].

Methicillin resistance was determined by the disk diffusion method testing oxacillin disk (30 *μ*g) on Mueller-Hinton agar supplemented with 2% sodium chloride.

### 2.4. Molecular Typing

Multiplex polymerase chain reaction (PCR) was applied to determine the SCC*mec* types according to a previous method described by Oliveira and de Lencastre [11].* pvl* genes (*luk*S-PV,* luk*F-PV) were detected by PCR as previously described [12].

### 2.5. PFGE

The isolates were genotyped by pulsed-field gel electrophoresis (PFGE) using the restriction enzyme* Sma*I according to the method previously described [13]. Pulsotypes findings were interpreted according to the criteria proposed by Tenover et al. [14]. The patterns were designated by capital letter. A chromosomal DNA digest of* S. aureus* strain NCTC 8325 was used as the reference strain.

### 2.6. MLST Typing

MLST typing was performed as described by Enright et al. [15]. Allelic profiles and sequence type (ST) were designated using the MLST database (http://www.mlst.net).

### 2.7. Nucleotide Sequence Accession Numbers

The GenBank accession numbers of staphylococcal gene sequences,* arcC*,* aroe*,* glpf*,* gmk*,* pta*,* tpi*, and* yqil*, determined in this study were, respectively, JF495119, JF495120, JF495121, JF495122, JF495123, JF495124, and JF495125.

## 3. Results

In this study we investigate 8 MRSA strains isolated from patients hospitalized in the pediatric department. All isolates were identified as MRSA strains by the determination of methicillin resistance using oxacillin disk diffusion method. Antimicrobial susceptibility showed that all isolates were susceptible to the majority of 22 antibiotics tested (see Section 2) with the exception of the beta-lactams (oxacillin, penicillin G, and cefoxitin). All strains were resistant to kanamycin and only two of them were resistant to erythromycin.

For all clinical isolates the detection of* mec*A gene and the identification of SCC*mec* type were performed by amplification from genomic DNA, using multiplex PCR method according to Oliveira and de Lencastre method [11]. For each isolate, 2 amplified fragments were obtained: a 162 bp fragment and a 342 bp fragment. These two PCR products correspond to the amplification of* mec*A gene and specific SCC*mec* type IV locus (*DCS*), respectively.

**Figure 1:**
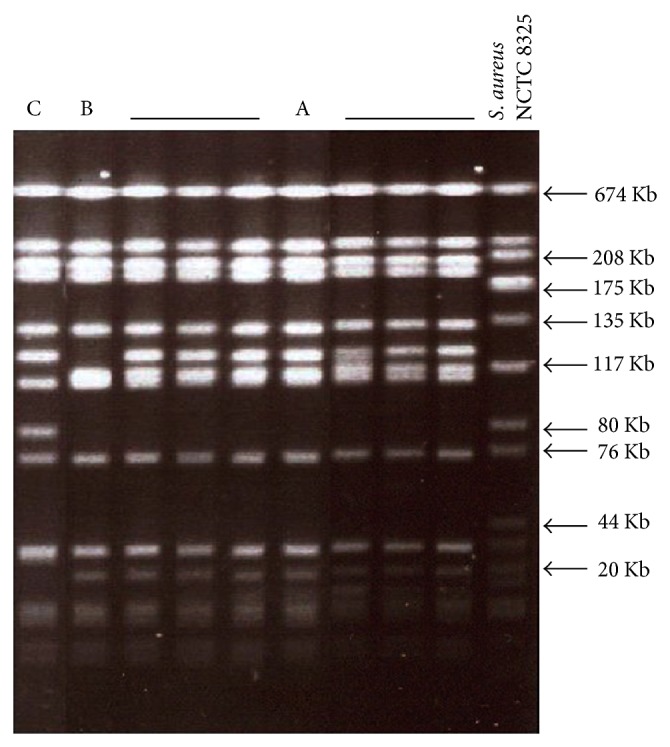
Representative PFGE patterns of MRSA strains isolated from children hospitalized in the pediatric department. A: pulsotype A; B: pulsotype B; C: pulsotype C; NCTC 8325: molecular weight marker.

Amplification of* pvl* genes (*luk*S-PV and* luk*F-PV) was performed also on the genomic DNA extracted from all strains. For each strain, the amplicon obtained has 433 bp; thus all MRSA strains harbor* pvl* genes. These results revealed that our clinical isolates have the peculiarities of CA-MRSA: susceptibility to the majority of antimicrobial agents and carrying SCC*mec* IV and* pvl* genes. To investigate the clonality of these CA-MRSA isolates, PFGE typing method was performed. PFGE pattern analysis demonstrated that they are distributed on three pulsotypes arbitrary designated A, B, and C (Figure 1). Six isolates carried the pulsotype A and two isolates carried the pulsotypes B and C, respectively. CA-MRSA strains were also characterized by multilocus sequencing of internal fragments of seven housekeeping genes (*arc*C,* aro*E,* glp*F,* gmk*,* pta*,* tpi*, and* yqil*). Nucleotide sequence analysis revealed that all isolates possess the same unique sequence type designated ST80.

## 4. Discussion and Conclusion

MRSA strain is known as a main cause of infections for children and young adults. MRSA strains investigated in this study are isolated from patients aged from 12 days to 18 months. Six children have been admitted with MRSA skin infections; hence, these data indicate that these infections were community-acquired but were not necessarily caused by community MRSA strains. However, the real site of MRSA acquisition is not readily determined because the community MRSA may designate MRSA colonization or a strain responsible for community infection detected in the community but not necessary acquired in the community. Two other ones are an 18-month-old child admitted with immunodeficiency and a 12-day-old child admitted with fever. They had cutaneous abscesses within 72 hours after their hospitalization. As referred to the definition of community MRSA infection, these isolates may be transmitted to these two patients from community.

Indeed, neonates are highly susceptible to MRSA colonization. CA-MRSA strains have been reported as a cause of colonization and infection in neonatal intensive care units in many countries [16, 17].

Furthermore, nasal carriage may be a possible explanation of the transmission of MRSA among these patients. The same observation was reported by Frazee et al. who considered that CA-MRSA is a common pathogen in cutaneous abscesses due to nasal carriage preceding infections [5]. It is interesting to note that newborns and young children, due to their immature immune systems, are easily infected by MRSA. Several similar studies reported that the majority of patients with MRSA infections were young children [6, 18]. For all strains antimicrobial resistance showed that they were resistant to oxacillin and susceptible to all non-beta-lactams. However the resistance to kanamycin and erythromycin has been also observed.

Molecular characterization of MRSA isolates by the identification of SCC*mec* type and the detection of* luk*S-PV and* luk*F-PV genes revealed that all strains harbored the SCC*mec* type IV and* pvl* genes. According to these results, our strains have been classified as community-acquired strains. Vandenesch et al. and Tenover et al. described SCC*mec* IV and* pvl* genes as markers for CA-MRSA [8, 19]. It has been reported that the cassette type IV and* pvl* genes have been found in some nosocomial MRSA strains [20–22].

PFGE analysis showed that CA-MRSA strains belonged to the same clone according to criteria of Tenover et al. [14]. MLST method revealed that all isolates have the same sequence type “ST80.” Full analyses of molecular typing results suggest that isolates belong to the CA-MRSA ST80 clone. In fact, this clone is being increasingly reported in the community worldwide and mainly detected in Europe [8].

Our CA-MRSA strains display the resistance to kanamycin and to erythromycin. This antibiotic resistance pattern seems to be different from European strains “ST80,” which were resistant to tetracycline and fusidic acid [23, 24].

MRSA is known as a nosocomial and a community pathogen. However, CA-MRSA has emerged within the hospital setting, posing a significant public health threat. So, what is most worrying is that these strains affect frequently newborns and young children and eventually cause potentially serious infections. In fact some MRSA epidemic clones have been reported to cause skins and soft tissue infections as well as severe diseases. Notably, “ST80” clone is recognized as a predominant clone in Europe, the United States, and Tunisia. This clone could become a health problem worldwide particularly that CA-MRSA strains were associated mainly with the presence of* pvl* genes which have an important impact on virulence. These observations urge emphasizing infection control measures to monitor the transmission of highly virulent CA-MRSA in our hospital.
